# HPV-driven oncogenesis—much more than the E6 and E7 oncoproteins

**DOI:** 10.1007/s13353-024-00883-y

**Published:** 2024-06-22

**Authors:** J. Janiszewska, M. Kostrzewska-Poczekaj, M. Wierzbicka, J. C. Brenner, M. Giefing

**Affiliations:** 1 Institute of Human Genetics, Polish Academy of Sciences, Strzeszynska 32, 60-479 Poznan, Poland; 2 Research & Development Centre, Regional Specialist Hospital Wroclaw, Wroclaw, Poland; 3 Faculty of Medicine, Wroclaw University of Science and Technology, Wroclaw, Poland; 4 Department of Otolaryngology - Head and Neck Surgery, University of Michigan Medical School, Ann Arbor, MI USA; 5 Rogel Cancer Center, University of Michigan Medical School, Ann Arbor, MI USA

**Keywords:** HPV 16, E6, E7, APOBEC, Viral miRNAs, Head and neck tumors

## Abstract

High-risk human papillomaviruses are well-established drivers of several cancer types including cervical, head and neck, penile as well as anal cancers. While the E6 and E7 viral oncoproteins have proven to be critical for malignant transformation, evidence is also beginning to emerge suggesting that both host pathways and additional viral genes may also be pivotal for malignant transformation. Here, we focus on the role of host APOBEC genes, which have an important role in molecular editing including in the response to the viral DNA and their role in HPV-driven carcinogenesis. Further, we also discuss data developed suggesting the existence of HPV-derived miRNAs in HPV + tumors and their potential role in regulating the host transcriptome. Collectively, while recent advances in these two areas have added complexity to the working model of papillomavirus-induced oncogenesis, these discoveries have also shed a light onto new areas of research that will be required to fully understand the process.

## Introduction

The human papillomavirus (HPV) is an 8 kb double-stranded circular DNA virus that belongs to the large family of papillomaviruses (PVs). Currently, there are known more than 200 types of HPV but only a subgroup is predicted to cause cancer. These are classified by the International Agency for Research on Cancer (Wild et al. 2020) as high-risk types of HPV (16, 18, 31, 33, 35, 39, 45, 51, 52, 56, 58, 59, 63, 73, and 82) and probable cancer-related types (26, 53, and 66). The low-risk viruses can cause warts (1 and 2) or benign changes within genital organs (6, 11, 40, 42, 43, 44, 54, 61, 70, 81) with no or poorly proven relation to human pathology (Clifford et al. 2003; Muñoz et al. 2003; Burd 2003; Kiwerska et al. 2019). The high-risk viruses are well known for cervical cancer pathogenesis where almost 100% of the tumors are attributed to the oncogenic activity of the HPV 16 and 18. However, HPV-related tumor pathogenesis is observed also in other sites like the anus (90% of cases), tumors of the vagina or penis (50–75% of cases), and the head and neck region (about 10% of cases) (de Martel et al. 2020; Serrano et al. 2018; Wierzbicka et al. 2021). The NIH estimates that worldwide, high-risk HPV infections cause about 5% of all cancers emphasizing on one hand the importance of papillomaviruses in the pathogenesis of human tumors and on the other the relevance of HPV vaccination („https://www.cancer.gov/”, n.d.).

## HPV 16-driven oncogenesis

### Canonical oncogenic mechanism that involves the E5, E6, and E7 oncoproteins

**Fig. 1:**
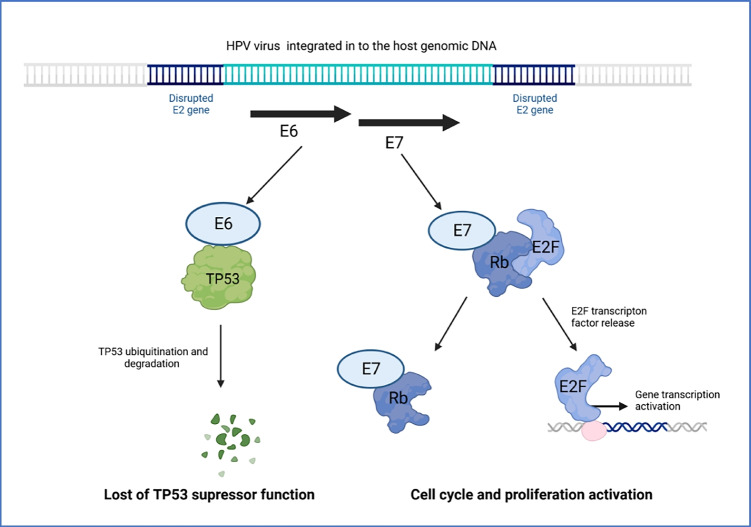
Oncogenic activity of the E6 and E7 proteins encoded by HPV 16 (based on Janicek and Averette (2001) and edited)

The main mechanism of HPV 16 and 18-driven oncogenesis is already well known, and for its explanation, Harald zur Hausen was awarded the Nobel Prize in Medicine in 2008 (Dürst et al. 1983; Boshart et al. 1984). The main players in this process are the oncoproteins E6 and E7 encoded by the viral genome (Münger et al. 1989). The expression of the E6 and E7 proteins is initiated after infection from the episomal form albeit on low levels because of the inhibition by the viral E2 protein. The proteins become significantly higher expressed after integration of the virus into the host genome which requires the transition from the circular to linear form resulting in the disruption of the E2 gene (Janicek and Averette 2001). The E6 and E7 proteins interact with crucial proteins of the cellular machinery responsible for cell cycle regulation and tumor suppression (Z.-A.-S. Ghoreshi 2023). E6 binds the cellular TP53 protein of well-known suppressor function triggering its ubiquitination and subsequent degradation by the E6AP ligase activity (E6-associated protein ligase) (Scheffner et al. 1993). This leads in consequence to loss of the regulatory function of TP53 towards the cell cycle and reduced expression of proapoptotic proteins such as *BAX* which is physiologically mediated by TP53 (Martinez-Zapien et al. 2016; Fischer 2017; Ruttkay-Nedecky et al. 2013). On the other hand, the E7 protein interacts with the cellular RB1 protein leading to the release of the potent transcription factor E2F/DP that in the G1 phase is normally bound to RB1 and inactive. The viral protein mimics therefore the physiological conditions of the late G1 phase where RB1 is inactivated by phosphorylation what leads to E2F/DP release (Rubin 2013). The E2F/DP factor drives the transcription of several genes responsible for the G1 → S transition, accelerates the cell cycle, and leads to proliferation. Eventually, the activity of the oncoproteins triggers the process of oncogenesis (Fig. 1).

Several cancer-related pathways are involved in this process. PI3K/AKT/mTOR is a well-known oncogenic pathway induced by E6 and E7 in head and neck cancer (Molinolo et al. 2009). These viral oncoproteins are able to active Akt pathway via upstream regulators, such as RPTK (receptor protein tyrosine kinase) and PI3K (phosphoinositide 3-kinase) resulting in increased proliferation (Aguayo et al. 2023).

Furthermore, E6 and E7 oncoproteins activate the Wnt pathway causing the accumulation of β-catenin, which may increase transcription of cell proliferation genes. This effect may be associated with a decreased protein level of β-catenin degradation inducer—Siah-1 (Bello et al. 2015). Moreover, accumulation of nuclear β-catenin is found in human HPV-positive cancers (Shinohara et al. 2001).

Similarly, NOTCH is affected in HPV-infected cells. The influence of E6 oncoprotein on NOTCH pathway regulation occurs through disruption of the interaction of MAML (mastermind-like protein) and NICD proteins, a critical component of the Notch signaling pathway. This results in turn in the loss of NOTCH target gene expression, including *HEY* and *HES*, which function as potent transcriptional repressors (Das et al. 2021; Galloway and Laimins 2015).

Another interesting aspect of E6 oncogenicity is the ability to interact with cellular proteins containing PDZ domains, such as PSD-95/Dlg/ZO-1 and Scribble, via the PBM motif (PDZ domain binding motif), presents in the C-terminus of the protein. This interaction disrupts intercellular connections between epithelial cells leading to loss of cell polarity which is a known characteristic of neoplastic cells (Gardiol et al. 1999).

In addition to the oncogenic potential of the E6 and E7 proteins, viral integration into the host genome directly triggers genomic instability (Akagi et al. 2014). Moreover, the introduced structural abnormalities may result in the disruption of tumor suppressor genes in the host genome and possibly also upregulate the expression of oncogenes (Schmitz et al. 2012; Lu et al. 2014) and reviewed in Chen et al. (2017) thus altering the function of several tumor-related genes.

Interestingly, one of the factors associated with increased risk of HPV-related malignant transformation may derive from bacterial or viral infections which can trigger alterations in the E6 oncoprotein expression. In this regard, Szostek et al. showed that the presence of *Ureaplasma urealyticum*–stimulated E6 expression in SiHa cells derived from human cervical squamous cell carcinoma cell line (Szostek et al. 2014).

Although the most known HPV-associated oncoproteins are E6 and E7, the E5 oncoprotein also plays a role in carcinogenesis. Recent studies have confirmed the importance of E5 oncoprotein in the modulation of the immune system and cell transformation. E5 cooperates with E7 and E6 to fuel the development of malignant cells (de Freitas et al. 2017). E5 oncoprotein inhibits apoptosis by increasing the ubiquitination and proteasomal degradation of the pro-apoptotic protein BAX consequently promoting the accumulation of cells with mutations (Maufort et al. 2010). Moreover, as shown for HPV 18, E5 potentiates EGFR signaling leading to increased ERK/MAPK activity and via this pathway is implicated in cell cycle progression. An important function of E5 oncoprotein was also confirmed in the metastatic process and is mediated by the upregulation of the MET oncoprotein (Scott et al. 2018; Hemmat and Baghi 2018).

### The non-canonical oncogenic mechanism related to the function of APOBEC enzymes

Recent reports suggest that the canonical mechanism of HPV-driven oncogenesis is not the only mechanism by which the virus contributes to tumor formation. The non-canonical mechanism of viral (including HPV) oncogenicity is related to the function of the APOBEC—apolipoprotein B mRNA editing enzymes. These enzymes belong to the class of APOBEC3s (A3A, A3B, A3C, A3DE, A3F, A3G, A3H) deaminases which become activated in the cell by an interferon-mediated signal cascade triggered by sensing foreign nucleic acids by the protein machinery of the cell (Stenglein et al. 2010). Several proteins like TLR9 and DAI, AIM2, and RNA polymerase III have been identified to sense single-stranded and double-stranded DNA, respectively (Uematsu and Akira 2007; Takaoka et al. 2007; Bürckstümmer et al. 2009; Chiu et al. 2009). Innate immune sensors play a crucial role in recognizing viral nucleic acids, including those from HPV viruses. They enable a rapid immune response to infections by secreting interferon (IFN), regulating IFN-stimulated genes, and activation of the inflammasome complex. For example, the nuclear DNA sensor IFI16 detects single and double-strand DNA particles upon which it moves to the cytoplasm and activates the TBK1-IRF3 and the pro-inflammatory NFκB signaling pathway. Interestingly, it contributes further to IFN expression through interaction with STING1, a major regulator of the innate immune response. It has been recently shown that STING1 can impact virtually all aspects of tumorigenesis (Z. Ghoreshi et al. 2022; Samson and Ablasser 2022).

**Fig. 2:**
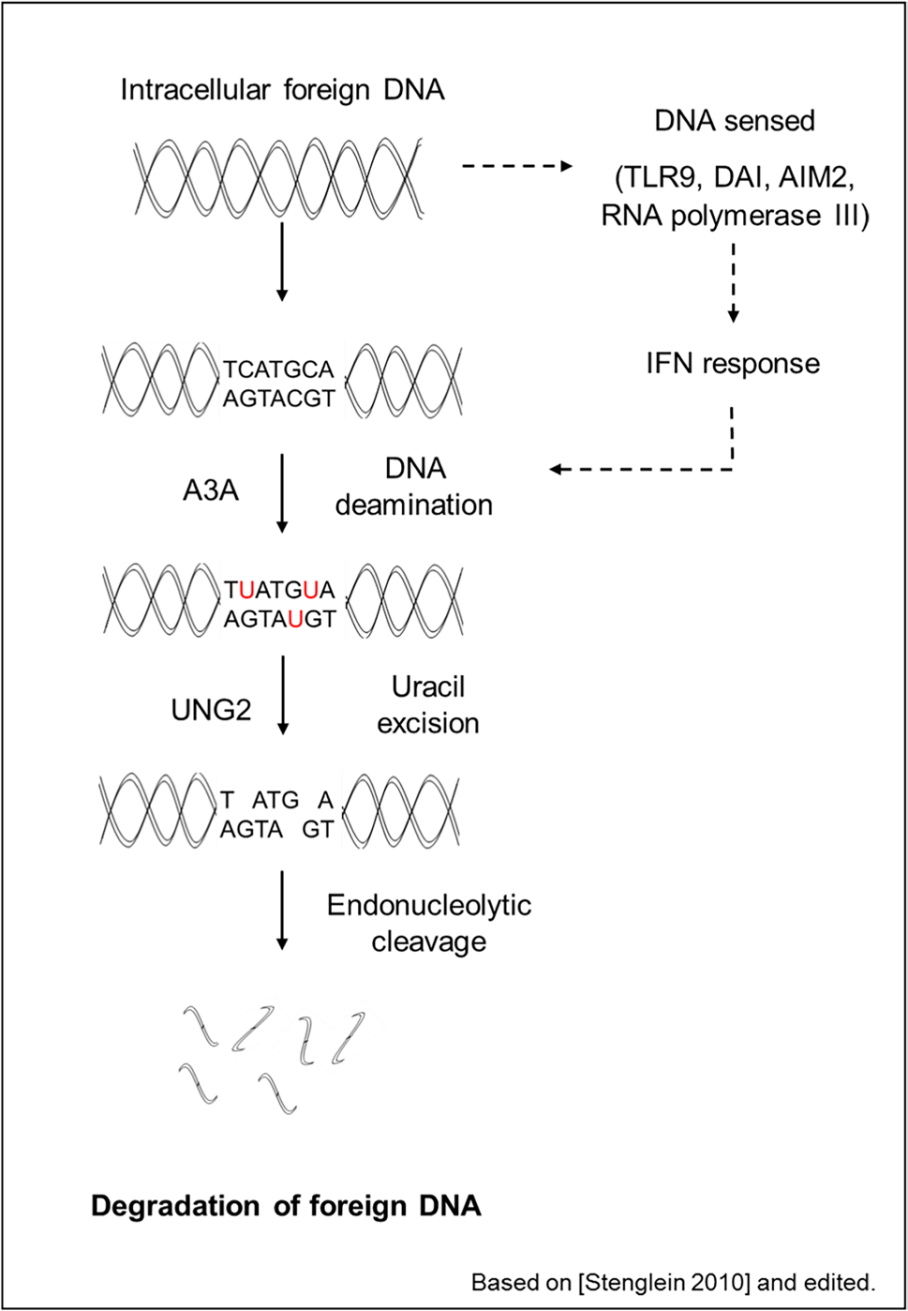
Degradation of foreign genetic material by the APOBEC deaminases (based on Stenglein et al. (2010) and edited)

The overexpressed APOBEC enzymes mediate the deamination of cytidines to uridines in the foreign DNA. As uridines are atypical DNA nucleosides, they are recognized by the UNG2 glycosylase and turned to abasic lesions leading to downstream degradation of the foreign DNA by uracil excision mechanism (Stenglein et al. 2010) (Fig. 2). In fact, it has been demonstrated that this class of enzymes edits HPV DNA in infected cells (Z. Wang et al. 2014). Together, this formulates a protective mechanism that prevents DNA transmission between species.

As the C → T transition is the predominant mutation in cancer genomes (Greenman et al. 2007), it allows to speculate that the virus-induced APOBEC enzymes might erroneously generate mutations in the genome of the host cell promoting malignant transformation (Suspène et al. 2011). In line with this presumption, the AID deaminase that is physiologically active during B-cell maturation in the germinal centers and mediates the process of somatic hypermutation (Maul and Gearhart 2010) was found involved in malignant transformation not only in B-cell lymphomas but also in nonlymphoid tumors (Okazaki et al. 2007). Moreover, the presence of typical mutations caused by deamination of cytosines introduced by APOBEC enzymes in a characteristic nucleotide context and thus having an APOBEC mutational signature has been demonstrated in genomes of multiple tumors (Alexandrov et al. 2013; Roberts et al. 2013). Interestingly, besides lymphoid neoplasms, APOBEC mutation signatures have been recognized in all HPV-related tumors including cervical cancers, esophageal cancers, or head and neck cancers suggesting an important role of this pathogenetics mechanism in these solid tumors. In fact, the frequent activating mutations E542K and E545K of the *PIK3CA* oncogene can be to a large extent attributed to the APOBEC-driven mutagenesis (Henderson et al. 2014) and reviewed in Chen et al. (2017). Faden et al. observed recently that APOBEC was the dominant mutational signature in somatic exomes in 79 HPV 16 + oropharyngeal squamous cell carcinoma cases analyzed. The authors assessed APOBEC mutational load in human and viral genomes concluding that there is a direct link in host and viral genome APOBEC-driven mutagenesis during infection. These provide an important link concerning APOBEC mutagenesis in host and virus and support a common mechanism driving APOBEC dysregulation (Faden et al. 2021).

It has been observed that in head and neck cancer patients, the APOBEC3B expression was significantly associated with treatment outcome (Janecka-Widła et al. 2021). In details, patients with high APOBEC3B expression detected in tumors had over two times higher risk of cancer progression than those with lower levels of this protein. In line with this observation, in the subgroup of HPV 16-positive HNSCC patients, those patients having tumors with low APOBEC3B expression survived significantly longer than those with high APOBEC3B expression (Janecka-Widła et al. 2021). Moreover, APOBEC3B overexpression differentiated HPV-negative low-grade oral epithelial dysplasia, which showed intermediate APOBEC3B expression, from oral cancers. The highest levels were found in grade II and III oral cancers suggesting that APOBEC3B may be used as a marker for advanced HPV-positive cancers (Argyris et al. 2021).

Thus, the erroneous functioning of the APOBEC enzymes induced by HPV infections may be an important mechanism of HPV oncogenicity, strictly related to the canonical mechanism described above. The continuous expression of oncoprotein E6 seems to be pivotal for *APOBEC3B* overexpression in HPV-associated cancers (Vieira et al. 2014). In line with this finding, Mori et al. demonstrated that E6-mediated degradation of the TP53 protein induces expression of the TEAD transcription factor, which is a positive regulator of *APOBEC3B* transcription (Mori et al. 2017). In addition, TEAD stabilizes the Yes-associated protein (YAP), an oncogenic component of the Hippo pathway (He et al. 2015).

The role of APOBEC enzymes in HPV-driven oncogenesis extends beyond the canonical mechanisms contributing to malignant transformation. Recent studies highlight the complexity and significance of APOBEC-related pathways in HPV-associated cancers; however, this non-canonical mechanism is likely secondary to the dominant mechanism based on HPV oncoproteins.

### The putative oncogenic mechanism related to viral miRNAs

Virus-encoded miRNAs not only show auto-regulatory function but have also the potential to interfere with various physiological functions of the infected cell. Viral miRNAs are found in several viral genomes including herpesviruses (Grundhoff and Sullivan 2011; Dass et al. 2023), adenoviruses (Xu et al. 2007), polyomaviruses (Sullivan et al. 2005), and also plant viruses (Sharma and Singh 2016). Such viral miRNAs have been identified in EBV (Epstein-Barr virus) and HCMV (cytomegalovirus) (Pfeffer et al. 2004; Kuzembayeva et al. 2012). It has been shown that the encoded miRNAs have important functions in the process of infection and malignant transformation. For example, in EBV-infected B-cells viral miRNAs reduce immunogenicity by suppressing proinflammatory cytokines such as IL-12. This results in reduced recruitment of CD4( +) effector T cells and subsequent impaired elimination of the infected B-cells (Tagawa et al. 2016). The expression of EBV miRNAs is also brought together with worse survival of EBV-associated gastric cancer (Kang et al. 2017). Furthermore, EBV-encoded miRNAs contribute to Endemic Burkitt lymphoma clinical presentation, progression, and poor outcome (Oduor et al. 2017). Moreover, viral miRNAs were shown to contribute to cell cycle deregulation by posttranscriptional silencing of cell cycle-related genes like *CCNE2* (cyclin E2), *BRCC3*, and *EID1* targeted by miR-US25-1 encoded by the HCMV genome (Grey et al. 2010).

**Fig. 3:**
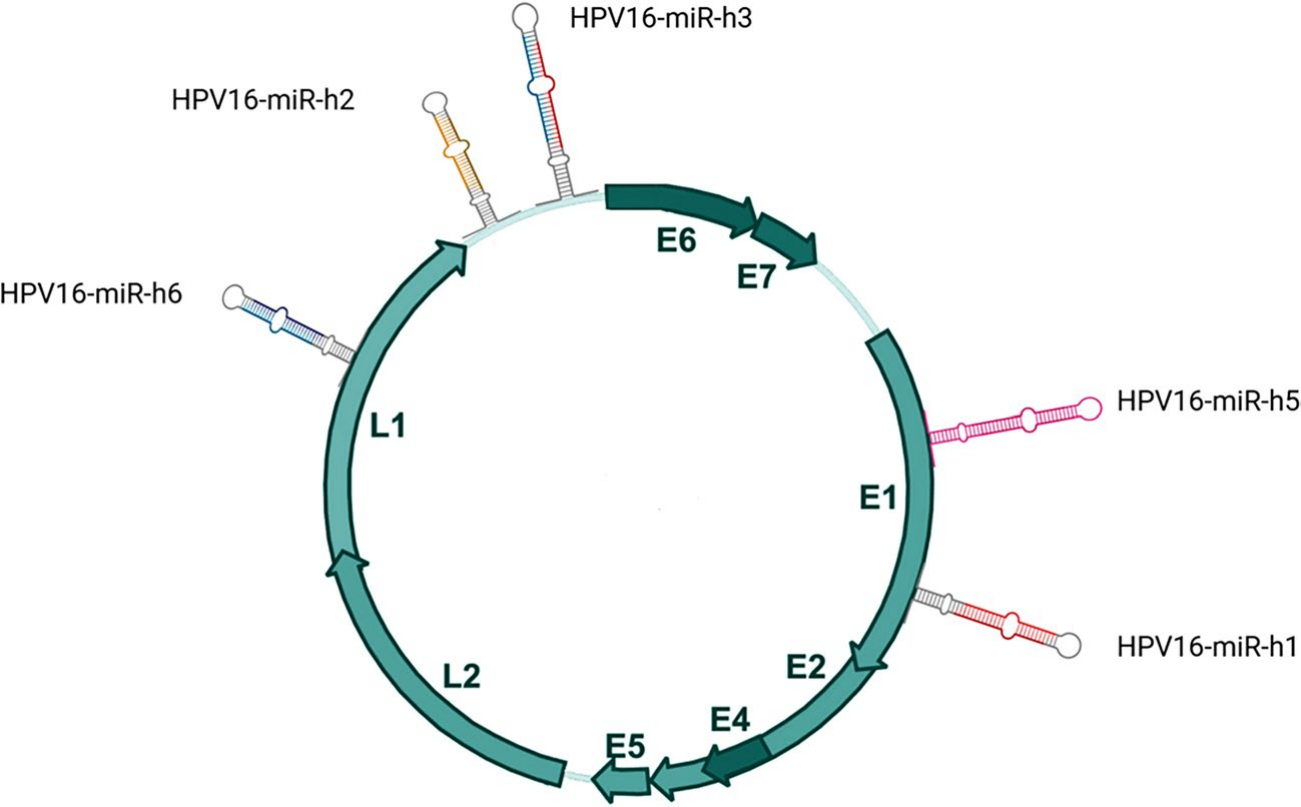
MiRNAs putatively encoded by the HPV 16 genome (based on Table 1)

In light of the presented findings, it is intriguing whether HPV genomes harbor miRNAs of similar functionalities. A decade ago, two manuscripts were published wherein the authors bioinformatically as well as experimentally demonstrate that the HPV 16 genome encodes beside protein coding also miRNA genes that are expressed, albeit on low level, in human cervical lesions (Qian et al. 2013; Virtanen et al. 2016). The authors identified five miRNAs HPV 16-miR-H1, 2, 3, 5, and 6 dispersed in the viral genome and encoded on the plus or minus strand (Table 1, Fig. 3). These findings have been further validated in parallel studies of other groups (Thakur 2015) and by bioinformatic analysis of NGS data (Weng et al. 2018). Several functions promoting viral infection have been implicated for these miRNAs; however, experimentally validated data demonstrating the viral miRNA:host mRNA interaction is still lacking. Viral HPV 16-miR-H1-1 and miR-H2-1 were predicted to target several genes involved in immune system regulation and development. HPV 16-miR-H1-1 via putative targeting of *BCL11A*, *CHD7*, *ITGAM*, *RAG1*, and *TCEA1* genes is able to inhibit T-cell activation leading to the inhibition of immune system development. Similarly, miR-H2-1 potentially inhibits genes involved in T-cell activation like *PKNOX1*, *SP3*, and *XRCC4* as well as immune system development (*JAK2*, *PKNOX1*, *SP3*, *XRCC4*, *FOXP1*) (Qian et al. 2013). Also, typical cancer-related tumor-suppressive genes are predicted targets of H1 including *CAV2*, *PTEN*, and *SEMA3F* (Qian et al. 2013). The same holds true for the other delineated HPV miRNAs as reviewed by Gallo et al. (Gallo et al. 2020). Gutiérrez et al. identified approximately 900 target genes potentially regulated by HPV-encoded miRNAs. Within this cohort, the largest sub-group, that is 15% of the genes, was involved in cell proliferation followed by regulation of ribosomes (11%) and translation (11%) (Gutiérrez et al. 2018).

**Table 1:** Putative HPV 16 (reference genome NC_001526.2)–encoded miRNAs and their target genes (Qian et al. 2013; Li et al. 2008; Qureshi et al. 2014)

miRNA	VMR_ID	Mature sequence	Length	miRDB predicted top targets (target score ≥ 98)	miRDB predicted top target genes (target score ≥ 98)
hpv16-miR-h1	VMR_0262	aguguaugagcuuaaugauaa	21	6	*ABO, TBX15, VPS13C, IGF1R, FBXL20, XKR4*
hpv16-miR-h2	VMR_0263	auguguaacccaaaacgguuug	22	3	*SLC10A7, VGLL3, PLEKHH3*
hpv16-miR-h3	VMR_0264	caacugaucucuacuguua	19	2	*QKI, MINDY2*
hpv16-miR-h5	VMR_0265	guaaagcauagaccauug	18	33	*MYT1L, ETNK1, ZBTB41, ACSL4, BMPR2, DCLK1, REST, QKI, ZNF704, GABRA1, FSD1L, TNRC6C, PPM1A, WWTR1, DCUN1D1, DCBLD2, SPRED1, CACNA1E, ZSWIM6, KAT2B, PHF3, RAB3C, GABPB1, HSPA4, SYNE2, LRP6, CDC27, GLS, WNK3, LYPLA1, DUT, CNTN1, ROCK2*
hpv16-miR-h6	VMR_0266	aucaacaacaguaacaaa	18	12	*ACSL3, ARID4B, ZBTB11, JMJD1C, GCNT1, CREBZF, SMAD2, RFX7, TOMM70, DCAF5, TNKS2, NMU*

Therefore, the cited literature data allow to carefully speculate that the putative HPV-encoded miRNAs, if real, might be involved in three crucial processes in the infected cell. That is (I) immune escape, (II) deregulation of cell cycle and attenuation of tumor suppressor genes leading to increased proliferation, and (III) takeover of the control of the cell’s transcriptional machinery. However, it needs to be stressed that the putative miRNA-driven oncogenic mechanism has a significantly inferior role compared to the function of the oncogenic virus-encoded proteins. Moreover, the functional validation of many of these findings is lacking; the proposed mechanism is speculative and not supported by direct target validation.

### Controversies

Although several studies reported the identification of HPV-encoded miRNAs, mainly in human cervical lesions as reviewed before, other authors came to opposite conclusions. Wang et al. by cloning and sequencing of a small RNA library from the HPV 16 + CaSki cell line identified 174 host miRNAs but none of the reported HPV 16 miRNAs and therefore concluded that the HPV 16 virus in the analyzed cell line does not express viral miRNAs (X. Wang et al. 2008). Similarly, Cai et al. reported no viral miRNAs to be expressed from the HPV 31 genome, and Lui et al. reported the lack of HPV-encoded miRNAs in direct sequencing of HPV-positive cervical cancer cell lines (Lui et al. 2007; Cai et al. 2006).

Interestingly, Chirayil et al. developed an approach based on a library of overlapping genomic segments from an analyzed genome subcloned behind a RNA polymerase II promoter resulting in forced expression of the putative miRNAs (miDGE—miRNA discovery by forced genomic expression) (Chirayil et al. 2018). Thereafter, miRNA candidates were identified using small RNA sequencing and downstream assays. Using this approach, the authors screened 73 different PV genomes (most were human-PVs) and identified five viral miRNA candidates, 1 in HPV 17, 1 in HPV 37, 1 in HPV 41, and 2 in FcPV1. However, no miRNA candidates were identified in the genomes of high-risk HPV 16, 18, and others. The authors further screened transcriptomes of cervical carcinomas deposited in The Cancer Genome Atlas (TCGA) (Cancer Genome Atlas Research Network et al. 2017), but similarly did not observe expression of any miRNA from the high-risk HPV genomes (Chirayil et al. 2018). Another argument against the existence of HPV-encoded miRNAs is the fact that none of the predicted top putative mRNA targets suggested in the cited studies like *BCL11A*, *ITGAM*, *RAG1*, *CAV2*, *PTEN*, and *SEMA3F* was identified in the miRNA target search using miRDB performed by us **(**Table 1**)** thus showing large inconsistencies between studies.

Altogether, the reviewed findings raise several unanswered questions. What are the apparently HPV-derived sequences found by various groups? Why are the results of different researcher groups not reproduced? Might they derive from co-infection by other PVs or are they only inactive sequences found in the host genome that are remains from past infections? Assuming that these sequences do not encode functional miRNAs, why do they show such enrichment in potential targets encoding important tumor-related genes hardly possible by pure chance? Are these putative miRNAs expressed only at specific time points in the viral life cycle? Or why should high-risk HPVs, in contrast to several low-risk PVs and other viruses, lack miRNAs that facilitate the process of infection and immune evasion? What was the selective force during the evolutionary process? These and other questions need to be answered in further studies.

## Conclusions

The mechanism of HPV-related carcinogenesis is much more complex than initially assumed. Since the discovery of the viral oncoproteins in the 80 s of the twentieth century, we witnessed the accumulation of experimental data which have significantly expanded the picture of how the virus infects the cell, takes over the control of its transcription machinery, and eventually leads to tumor formation. In this process, the function of the virus-encoded oncoproteins is supported by triggered genomic instability, accelerated mutagenesis via the APOBEC enzymes, and perhaps also by yet not fully understood viral miRNA sequences.

## References

[CR1] Aguayo F, Perez-Dominguez F, Osorio JC, Oliva C, Calaf GM (2023) PI3K/AKT/mTOR signaling pathway in HPV-driven head and neck carcinogenesis: therapeutic implications. Biology 12(5):672. 10.3390/biology12050672 37237486 10.3390/biology12050672 PMC10215516

[CR2] Akagi K, Li J, Broutian TR, Padilla-Nash H, Xiao W, Jiang B, Rocco JW et al (2014) Genome-wide analysis of HPV integration in human cancers reveals recurrent, focal genomic instability. Genome Res 24(2):185–99. 10.1101/gr.164806.113 24201445 10.1101/gr.164806.113 PMC3912410

[CR3] Alexandrov LB, Nik-Zainal S, Wedge DC, Aparicio SAJR, Behjati S, Biankin AV, Bignell GR et al (2013) Signatures of mutational processes in human cancer. Nature 500(7463):415–21. 10.1038/nature12477 23945592 10.1038/nature12477 PMC3776390

[CR4] Argyris PP, Wilkinson PE, Jarvis MC, Magliocca KR, Patel MR, Vogel RI, Gopalakrishnan R, Koutlas IG, Harris RS (2021) Endogenous APOBEC3B overexpression characterizes HPV-positive and HPV-negative oral epithelial dysplasias and head and neck cancers. Mod Pathol Off J U S Can Acad Pathol Inc 34(2):280–90. 10.1038/s41379-020-0617-x 10.1038/s41379-020-0617-x PMC8261524 32632179

[CR5] Bello JOM, Nieva LO, Paredes AC, Gonzalez AMF, Zavaleta LR, Lizano M (2015) Regulation of the Wnt/β-catenin signaling pathway by human papillomavirus E6 and E7 oncoproteins. Viruses 7(8):4734–55. 10.3390/v7082842 26295406 10.3390/v7082842 PMC4576203

[CR6] Boshart M, Gissmann L, Ikenberg H, Kleinheinz A, Scheurlen W, zur Hausen H (1984) A new type of papillomavirus DNA, its presence in genital cancer biopsies and in cell lines derived from cervical cancer. EMBO J 3(5):1151–57. 10.1002/j.1460-2075.1984.tb01944.x 6329740 10.1002/j.1460-2075.1984.tb01944.x PMC557488

[CR7] Bürckstümmer T, Baumann C, Blüml S, Dixit E, Dürnberger G, Jahn H, Planyavsky M et al (2009) An orthogonal proteomic-genomic screen identifies AIM2 as a cytoplasmic DNA sensor for the inflammasome. Nat Immunol 10(3):266–72. 10.1038/ni.1702 19158679 10.1038/ni.1702

[CR8] Burd EM (2003) Human papillomavirus and cervical cancer. Clin Microbiol Rev 16(1):1–17. 10.1128/CMR.16.1.1-17.2003 12525422 10.1128/CMR.16.1.1-17.2003 PMC145302

[CR9] Cai X, Li G, Laimins LA, Cullen BR (2006) Human papillomavirus genotype 31 does not express detectable microRNA levels during latent or productive virus replication. J Virol 80(21):10890–93. 10.1128/JVI.01175-06 17041229 10.1128/JVI.01175-06 PMC1641796

[CR10] Cancer Genome Atlas Research Network, Albert Einstein College of Medicine, Analytical Biological Services, Barretos Cancer Hospital, Baylor College of Medicine, Beckman Research Institute of City of Hope, Buck Institute for Research on Aging et al (2017) Integrated genomic and molecular characterization of cervical cancer. Nature 543(7645):378–84. 10.1038/nature21386 28112728 10.1038/nature21386 PMC5354998

[CR11] Chen L, Qiu X, Zhang N, Wang Y, Wang M, Li D, Wang L, Du Y (2017) APOBEC-mediated genomic alterations link immunity and viral infection during human papillomavirus-driven cervical carcinogenesis. Biosci Trends 11(4):383–88. 10.5582/bst.2017.01103 28717061 10.5582/bst.2017.01103

[CR12] Chirayil R, Kincaid RP, Dahlke C, Kuny CV, Dälken N, Spohn M, Lawson B, Grundhoff A, Sullivan CS (2018) Identification of virus-encoded microRNAs in divergent papillomaviruses. PLoS Pathog 14(7):e1007156. 10.1371/journal.ppat.1007156 30048533 10.1371/journal.ppat.1007156 PMC6062147

[CR13] Chiu Y-H, Macmillan JB, Chen ZJ (2009) RNA polymerase III detects cytosolic DNA and induces type I interferons through the RIG-I pathway. Cell 138(3):576–91. 10.1016/j.cell.2009.06.015 19631370 10.1016/j.cell.2009.06.015 PMC2747301

[CR14] Clifford GM, Smith JS, Plummer M, Muñoz N, Franceschi S (2003) Human papillomavirus types in invasive cervical cancer worldwide: a meta-analysis. Br J Cancer 88(1):63–73. 10.1038/sj.bjc.6600688 12556961 10.1038/sj.bjc.6600688 PMC2376782

[CR15] Das T, Zhong R, Spiotto MT (2021) Notch signaling and human papillomavirus-associated oral tumorigenesis. Adv Exp Med Biol 1287:105–22. 10.1007/978-3-030-55031-8_8 33034029 10.1007/978-3-030-55031-8_8 PMC7751007

[CR16] Dass D, Dhotre K, Chakraborty M, Nath A, Banerjee A, Bagchi P, Mukherjee A (2023) miRNAs in herpesvirus infection: powerful regulators in small packages. Viruses 15(2):429. 10.3390/v15020429 36851643 10.3390/v15020429 PMC9965283

[CR17] de Freitas AC, de Oliveira THA, Barros MR, Venuti A (2017) hrHPV E5 oncoprotein: immune evasion and related immunotherapies. J Exp Clin Cancer Res: CR 36(1):71. 10.1186/s13046-017-0541-1 28545552 10.1186/s13046-017-0541-1 PMC5445378

[CR18] de Martel C, Georges D, Bray F, Ferlay J, Clifford GM (2020) Global burden of cancer attributable to infections in 2018: a worldwide incidence analysis. Lancet Glob Health 8(2):e180-90. 10.1016/S2214-109X(19)30488-7 31862245 10.1016/S2214-109X(19)30488-7

[CR19] Dürst M, Gissmann L, Ikenberg H, zur Hausen H (1983) A papillomavirus DNA from a cervical carcinoma and its prevalence in cancer biopsy samples from different geographic regions. Proc Natl Acad Sci USA 80(12):3812–15. 10.1073/pnas.80.12.3812 6304740 10.1073/pnas.80.12.3812 PMC394142

[CR20] Faden DL, Kuhs KAL, Lin M, Langenbucher A, Pinheiro M, Yeager M, Cullen M et al (2021) APOBEC mutagenesis is concordant between tumor and viral genomes in HPV-positive head and neck squamous cell carcinoma. Viruses 13(8):1666. 10.3390/v13081666 34452530 10.3390/v13081666 PMC8402723

[CR21] Fischer M (2017) Census and evaluation of P53 target genes. Oncogene 36(28):3943–3956. 10.1038/onc.2016.502 28288132 10.1038/onc.2016.502 PMC5511239

[CR22] Gallo A, Miceli V, Bulati M, Iannolo G, Contino F, Conaldi PG (2020) Viral miRNAs as active players and participants in tumorigenesis. Cancers 12(2):358. 10.3390/cancers12020358 32033193 10.3390/cancers12020358 PMC7072176

[CR23] Galloway DA, Laimins LA (2015) Human papillomaviruses: shared and distinct pathways for pathogenesis. Curr Opin Virol 14(październik):87–92. 10.1016/j.coviro.2015.09.001 26398222 10.1016/j.coviro.2015.09.001 PMC4628885

[CR24] Gardiol D, Kühne C, Glaunsinger B, Lee SS, Javier R, Banks L (1999) Oncogenic human papillomavirus E6 proteins target the discs large tumour suppressor for proteasome-mediated degradation. Oncogene 18(40):5487–96. 10.1038/sj.onc.1202920 10523825 10.1038/sj.onc.1202920

[CR25] Ghoreshi Z-A-S (2023) The role of DNA viruses in human cancer. Cancer Inform 22:11769351231154186. 10.1177/11769351231154186 37363356 10.1177/11769351231154186 PMC10286548

[CR26] Ghoreshi Z-A, Nakhaee M, Samie M, Zak MS, Arefini N (2022) Innate immune sensors for detecting nucleic acids during infection. J Lab Med. 10.1515/labmed-2021-0173

[CR27] Greenman C, Stephens P, Smith R, Dalgliesh GL, Hunter C, Bignell G, Davies H et al (2007) Patterns of somatic mutation in human cancer genomes. Nature 446(7132):153–58. 10.1038/nature05610 17344846 10.1038/nature05610 PMC2712719

[CR28] Grey F, Tirabassi R, Meyers H, Wu G, McWeeney S, Hook L, Nelson JA (2010) A viral microRNA down-regulates multiple cell cycle genes through mRNA 5’UTRs. PLoS Pathog 6(6):e1000967. 10.1371/journal.ppat.1000967 20585629 10.1371/journal.ppat.1000967 PMC2891821

[CR29] Grundhoff A, Sullivan CS (2011) Virus-encoded microRNAs. Virology 411(2):325–43. 10.1016/j.virol.2011.01.002 21277611 10.1016/j.virol.2011.01.002 PMC3052296

[CR30] Gutiérrez DA, Varela-Ramírez A, Rodríguez-Esquivel M, Mendoza-Rodríguez MG, Ayala-Sumuano JT, Pineda D, Garrido-Guerrero E et al (2018) Predicting human miRNA-like sequences within human papillomavirus genomes. Arch Med Res 49(5):323–34. 10.1016/j.arcmed.2018.10.008 30401587 10.1016/j.arcmed.2018.10.008

[CR31] He C, Mao D, Hua G, Lv X, Chen X, Angeletti PC, Dong J et al (2015) The Hippo/YAP pathway interacts with EGFR signaling and HPV oncoproteins to regulate cervical cancer progression. EMBO Mol Med 7(11):1426–49. 10.15252/emmm.201404976 26417066 10.15252/emmm.201404976 PMC4644376

[CR32] Hemmat N, Baghi HB (2018) Human papillomavirus E5 protein, the undercover culprit of tumorigenesis. Infect Agents Cancer 13:31. 10.1186/s13027-018-0208-3 10.1186/s13027-018-0208-3 PMC6230221 30455726

[CR33] Henderson S, Chakravarthy A, Su X, Boshoff C, Fenton TR (2014) APOBEC-mediated cytosine deamination links PIK3CA helical domain mutations to human papillomavirus-driven tumor development. Cell Rep 7(6):1833–41. 10.1016/j.celrep.2014.05.012 24910434 10.1016/j.celrep.2014.05.012

[CR34] https://www.cancer.gov/ (n.d.)

[CR35] Janecka-Widła A, Majchrzyk K, Mucha-Małecka A, Biesaga B (2021) EGFR/PI3K/Akt/mTOR pathway in head and neck squamous cell carcinoma patients with different HPV status. Pol J Pathol: Off J Pol Soc Pathol 72(4):296–314. 10.5114/pjp.2021.113073 10.5114/pjp.2021.113073 35142162

[CR36] Janicek MF, Averette HE (2001) Cervical cancer: prevention, diagnosis, and therapeutics. CA: Cancer J Clin 51(2):92–114. 10.3322/canjclin.51.2.92. quiz 115–18 11577486 10.3322/canjclin.51.2.92

[CR37] Kang BW, Choi Y, Kwon OK, Lee SS, Chung HY, Yu W, Bae HI et al (2017) High level of viral microRNA-BART20–5p expression is associated with worse survival of patients with epstein-barr virus-associated gastric cancer. Oncotarget 8(9):14988–94. 10.18632/oncotarget.14744 28122341 10.18632/oncotarget.14744 PMC5362460

[CR38] Kiwerska K, Jozefiak A, Markowska J, Kedzia W, Jackowska J, Wierzbicka M (2019) Oral-genital human papillomavirus infection in Polish couples: frequent detection of HPV 42. BMC Infect Dis 19(1):122. 10.1186/s12879-018-3645-0 30727957 10.1186/s12879-018-3645-0 PMC6364387

[CR39] Kuzembayeva M, Chiu Y-F, Sugden B (2012) Comparing proteomics and RISC immunoprecipitations to identify targets of Epstein-Barr viral miRNAs. PloS One 7(10):e47409. 10.1371/journal.pone.0047409 23091622 10.1371/journal.pone.0047409 PMC3472983

[CR40] Li S-C, Shiau C-K, Lin W-C (2008) Vir-Mir Db: prediction of viral microRNA candidate hairpins. Nucleic Acids Res 36(Database issue):D184-189. 10.1093/nar/gkm610 17702763 10.1093/nar/gkm610 PMC2238904

[CR41] Lu X, Lin Q, Lin M, Duan P, Ye L, Chen J, Chen X, Zhang L, Xue X (2014) Multiple-integrations of HPV16 genome and altered transcription of viral oncogenes and cellular genes are associated with the development of cervical cancer. PloS One 9(7):e97588. 10.1371/journal.pone.0097588 24992025 10.1371/journal.pone.0097588 PMC4081011

[CR42] Lui W-O, Pourmand N, Patterson BK, Fire A (2007) Patterns of known and novel small RNAs in human cervical cancer. Cancer Res 67(13):6031–43. 10.1158/0008-5472.CAN-06-0561 17616659 10.1158/0008-5472.CAN-06-0561

[CR43] Martinez-Zapien D, Ruiz FX, Poirson J, Mitschler A, Ramirez J, Forster A, Cousido-Siah A et al (2016) Structure of the E6/E6AP/P53 complex required for HPV-mediated degradation of P53. Nature 529(7587):541–45. 10.1038/nature16481 26789255 10.1038/nature16481 PMC4853763

[CR44] Maufort JP, Shai A, Pitot HC, Lambert PF (2010) A role for HPV16 E5 in cervical carcinogenesis. Cancer Res 70(7):2924–31. 10.1158/0008-5472.CAN-09-3436 20332225 10.1158/0008-5472.CAN-09-3436 PMC2848882

[CR45] Maul RW, Gearhart PJ (2010) AID and somatic hypermutation. Adv Immunol 105:159–91. 10.1016/S0065-2776(10)05006-6 20510733 10.1016/S0065-2776(10)05006-6 PMC2954419

[CR46] Molinolo AA, Amornphimoltham P, Squarize CH, Castilho RM, Patel V, Gutkind JS (2009) Dysregulated molecular networks in head and neck carcinogenesis. Oral Oncol 45(4–5):324–34. 10.1016/j.oraloncology.2008.07.011 18805044 10.1016/j.oraloncology.2008.07.011 PMC2743485

[CR47] Mori S, Takeuchi T, Ishii Y, Yugawa T, Kiyono T, Nishina H, Kukimoto I (2017) Human papillomavirus 16 E6 upregulates APOBEC3B via the TEAD transcription factor. J Virol 91(6):e02413-16. 10.1128/JVI.02413-16 28077648 10.1128/JVI.02413-16 PMC5331809

[CR48] Münger K, Phelps WC, Bubb V, Howley PM, Schlegel R (1989) The E6 and E7 genes of the human papillomavirus type 16 together are necessary and sufficient for transformation of primary human keratinocytes. J Virol 63(10):4417–21. 10.1128/JVI.63.10.4417-4421.1989 2476573 10.1128/jvi.63.10.4417-4421.1989 PMC251060

[CR49] Muñoz N, Bosch FX, de Sanjosé S, Herrero R, Castellsagué X, Shah KV, Snijders PJF, Meijer CJLM, International Agency for Research on Cancer Multicenter Cervical Cancer Study Group (2003) Epidemiologic classification of human papillomavirus types associated with cervical cancer. N Engl J Med 348(6):518–27. 10.1056/NEJMoa021641 12571259 10.1056/NEJMoa021641

[CR50] Oduor CI, Movassagh M, Kaymaz Y, Chelimo K, Otieno J, Ong’echa JM, Moormann AM, Bailey JA (2017) Human and Epstein-Barr virus miRNA profiling as predictive biomarkers for endemic Burkitt lymphoma. Front Microbiol 8:501. 10.3389/fmicb.2017.00501 28400759 10.3389/fmicb.2017.00501 PMC5368269

[CR51] Okazaki I-m, Kotani A, Honjo T (2007) Role of AID in tumorigenesis. Adv Immunol 94:245–73. 10.1016/S0065-2776(06)94008-5 17560277 10.1016/S0065-2776(06)94008-5

[CR52] Pfeffer S, Zavolan M, Grässer FA, Chien M, Russo JJ, Ju J, John B et al (2004) Identification of virus-encoded microRNAs. Science (New York, N.Y.) 304(5671):734–36. 10.1126/science.1096781 15118162 10.1126/science.1096781

[CR53] Qian K, Pietilä T, Rönty M, Michon F, Frilander MJ, Ritari J (2013) Identification and validation of human papillomavirus encoded microRNAs. PLoS One 8(7):e70202 10.1371/journal.pone.0070202 PMC3728184 23936163

[CR54] Qureshi A, Thakur N, Monga I, Thakur A, Kumar M (2014) VIRmiRNA: a comprehensive resource for experimentally validated viral miRNAs and their targets. Database: J Biol Databases Curation 2014:bau103. 10.1093/database/bau103 10.1093/database/bau103 PMC4224276 25380780

[CR55] Roberts SA, Lawrence MS, Klimczak LJ, Grimm SA, Fargo D, Stojanov P, Kiezun A et al (2013) An APOBEC cytidine deaminase mutagenesis pattern is widespread in human cancers. Nat Genet 45(9):970–76. 10.1038/ng.2702 23852170 10.1038/ng.2702 PMC3789062

[CR56] Rubin SM (2013) Deciphering the retinoblastoma protein phosphorylation code. Trends Biochem Sci 38(1):12–19. 10.1016/j.tibs.2012.10.007 23218751 10.1016/j.tibs.2012.10.007 PMC3529988

[CR57] Ruttkay-Nedecky B, Jimenez Jimenez AM, Nejdl L, Chudobova D, Gumulec J, Masarik M, Adam V, Kizek R (2013) Relevance of infection with human papillomavirus: the role of the P53 tumor suppressor protein and E6/E7 zinc finger proteins (Review). Int J Oncol 43(6):1754–62. 10.3892/ijo.2013.2105 24045364 10.3892/ijo.2013.2105

[CR58] Samson N, Ablasser A (2022) The cGAS–STING pathway and cancer. Nat Cancer 3:1452–1463. 10.1038/s43018-022-00468-w 10.1038/s43018-022-00468-w 36510011

[CR59] Scheffner M, Huibregtse JM, Vierstra RD, Howley PM (1993) The HPV-16 E6 and E6-AP complex functions as a ubiquitin-protein ligase in the ubiquitination of P53. Cell 75(3):495–505. 10.1016/0092-8674(93)90384-3 8221889 10.1016/0092-8674(93)90384-3

[CR60] Schmitz M, Driesch C, Beer-Grondke K, Jansen L, Runnebaum IB, Dürst M (2012) Loss of gene function as a consequence of human papillomavirus DNA integration. Int J Cancer 131(5):E593-602. 10.1002/ijc.27433 22262398 10.1002/ijc.27433

[CR61] Scott ML, Coleman DT, Kelly KC, Carroll JL, Woodby B, Songock WK, Cardelli JA, Bodily JM (2018) Human papillomavirus type 16 E5-mediated upregulation of met in human keratinocytes. Virology 519(czerwiec):1–11. 10.1016/j.virol.2018.03.021 29609071 10.1016/j.virol.2018.03.021 PMC5971161

[CR62] Serrano B, Brotons M, Bosch FX, Bruni L (2018) Epidemiology and burden of HPV-related disease. Best Pract Res Clin Obstet Gynaecol 47(luty):14–26. 10.1016/j.bpobgyn.2017.08.006 29037457 10.1016/j.bpobgyn.2017.08.006

[CR63] Sharma N, Singh SK (2016) Implications of non-coding RNAs in viral infections. Rev Med Virol 26(5):356–68. 10.1002/rmv.1893 27401792 10.1002/rmv.1893

[CR64] Shinohara A, Yokoyama Y, Wan X, Takahashi Y, Mori Y, Takami T, Shimokawa K, Tamaya T (2001) Cytoplasmic/nuclear expression without mutation of Exon 3 of the beta-catenin gene is frequent in the development of the neoplasm of the uterine cervix. Gynecol Oncol 82(3):450–55. 10.1006/gyno.2001.6298 11520139 10.1006/gyno.2001.6298

[CR65] Stenglein MD, Burns MB, Li M, Lengyel J, Harris RS (2010) APOBEC3 proteins mediate the clearance of foreign DNA from human cells. Nat Struct Mol Biol 17(2):222–29. 10.1038/nsmb.1744 20062055 10.1038/nsmb.1744 PMC2921484

[CR66] Sullivan CS, Grundhoff AT, Tevethia S, Pipas JM, Ganem D (2005) SV40-encoded microRNAs regulate viral gene expression and reduce susceptibility to cytotoxic T cells. Nature 435(7042):682–86. 10.1038/nature03576 15931223 10.1038/nature03576

[CR67] Suspène R, Aynaud M-M, Guétard D, Henry M, Eckhoff G, Marchio A, Pineau P, Dejean A, Vartanian J-P, Wain-Hobson S (2011) Somatic hypermutation of human mitochondrial and nuclear DNA by APOBEC3 cytidine deaminases, a pathway for DNA catabolism. Proc Natl Acad Sci USA 108(12):4858–63. 10.1073/pnas.1009687108 21368204 10.1073/pnas.1009687108 PMC3064337

[CR68] Szostek S, Zawilińska B, Biernat-Sudolska M, Kopeć J, Kłeszcz E, Koprynia M, Rojek-Zakrzewska D, Kosz-Vnenchak M (2014) Differences in the expression of human papillomavirus type 16 (HPV-16) E6 oncogene mRNA in SiHa cell line inoculated with CMV, HSV or ureaplasmas. Folia Biol 62(1):73–78. 10.3409/fb62_1.73 10.3409/fb62_1.73 24745152

[CR69] Tagawa T, Albanese M, Bouvet M, Moosmann A, Mautner J, Heissmeyer V, Zielinski C et al (2016) Epstein-Barr viral miRNAs inhibit antiviral CD4+ T cell responses targeting IL-12 and peptide processing. J Exp Med 213(10):2065–80. 10.1084/jem.20160248 27621419 10.1084/jem.20160248 PMC5030804

[CR70] Takaoka A, Wang Z, Choi MK, Yanai H, Negishi H, Ban T, Lu Y et al (2007) DAI (DLM-1/ZBP1) is a cytosolic DNA sensor and an activator of innate immune response. Nature 448(7152):501–5. 10.1038/nature06013 17618271 10.1038/nature06013

[CR71] Thakur S (2015) Gynecologic and obstetric pathology. Mod Pathol 28(2):271–318. 10.1038/modpathol.2015.17

[CR72] Uematsu S, Akira S (2007) Toll-like receptors and type I interferons. J Biol Chem 282(21):15319–23. 10.1074/jbc.R700009200 17395581 10.1074/jbc.R700009200

[CR73] Vieira VC, Leonard B, White EA, Starrett GJ, Temiz NA, Lorenz LD, Lee D et al (2014) Human papillomavirus E6 triggers upregulation of the antiviral and cancer genomic DNA deaminase APOBEC3B. mBio 5(6):e02234-14. 10.1128/mBio.02234-14 25538195 10.1128/mBio.02234-14 PMC4278539

[CR74] Virtanen E, Pietilä T, Nieminen P, Qian K, Auvinen E (2016) Low expression levels of putative HPV encoded microRNAs in cervical samples. SpringerPlus 5(1):1856. 10.1186/s40064-016-3524-3 27818894 10.1186/s40064-016-3524-3 PMC5075338

[CR75] Wang X, Tang S, Le S-Y, Lu R, Rader JS, Meyers C, Zheng Z-M (2008) Aberrant expression of oncogenic and tumor-suppressive microRNAs in cervical cancer is required for cancer cell growth. PloS One 3(7):e2557. 10.1371/journal.pone.0002557 18596939 10.1371/journal.pone.0002557 PMC2438475

[CR76] Wang Z, Wakae K, Kitamura K, Aoyama S, Liu G, Koura M, Monjurul AM, Kukimoto I, Muramatsu M (2014) APOBEC3 deaminases induce hypermutation in human papillomavirus 16 DNA upon beta interferon stimulation. J Virol 88(2):1308–17. 10.1128/JVI.03091-13 24227842 10.1128/JVI.03091-13 PMC3911654

[CR77] Weng S-L, Huang K-Y, Weng JT-Y, Hung F-Y, Chang T-H, Lee T-Y (2018) Genome-wide discovery of viral microRNAs based on phylogenetic analysis and structural evolution of various human papillomavirus subtypes. Brief Bioinform 19(6):1102–14. 10.1093/bib/bbx046 28531277 10.1093/bib/bbx046

[CR78] Wierzbicka M, Klussmann JP, San Giorgi MR, Wuerdemann N, Dikkers FG (2021) Oral and laryngeal HPV infection: incidence, prevalence and risk factors, with special regard to concurrent infection in head, neck and genitals. Vaccine 39(17):2344–50. 10.1016/j.vaccine.2021.03.047 33812740 10.1016/j.vaccine.2021.03.047

[CR79] Wild CP, Weiderpass E, Stewart BW (2020) World cancer report: cancer research for cancer prevention. International Agency for Research on Cancer: Lyon, France,. 2020 39432694

[CR80] Xu N, Segerman B, Zhou X, Akusjärvi G (2007) Adenovirus virus-associated RNAII-derived small RNAs are efficiently incorporated into the Rna-induced silencing complex and associate with polyribosomes. J Virol 81(19):10540–49. 10.1128/JVI.00885-07 17652395 10.1128/JVI.00885-07 PMC2045446

